# Supplementary figures and images for: Analysis of multiple bacterial species and antibiotic classes reveals large variation in the association between seasonal antibiotic use and resistance
Source: PLoS Biol. 2022 Mar 9;20(3):e3001579. doi: 10.1371/journal.pbio.3001579 (PMC8936496; doi:10.1371/journal.pbio.3001579)

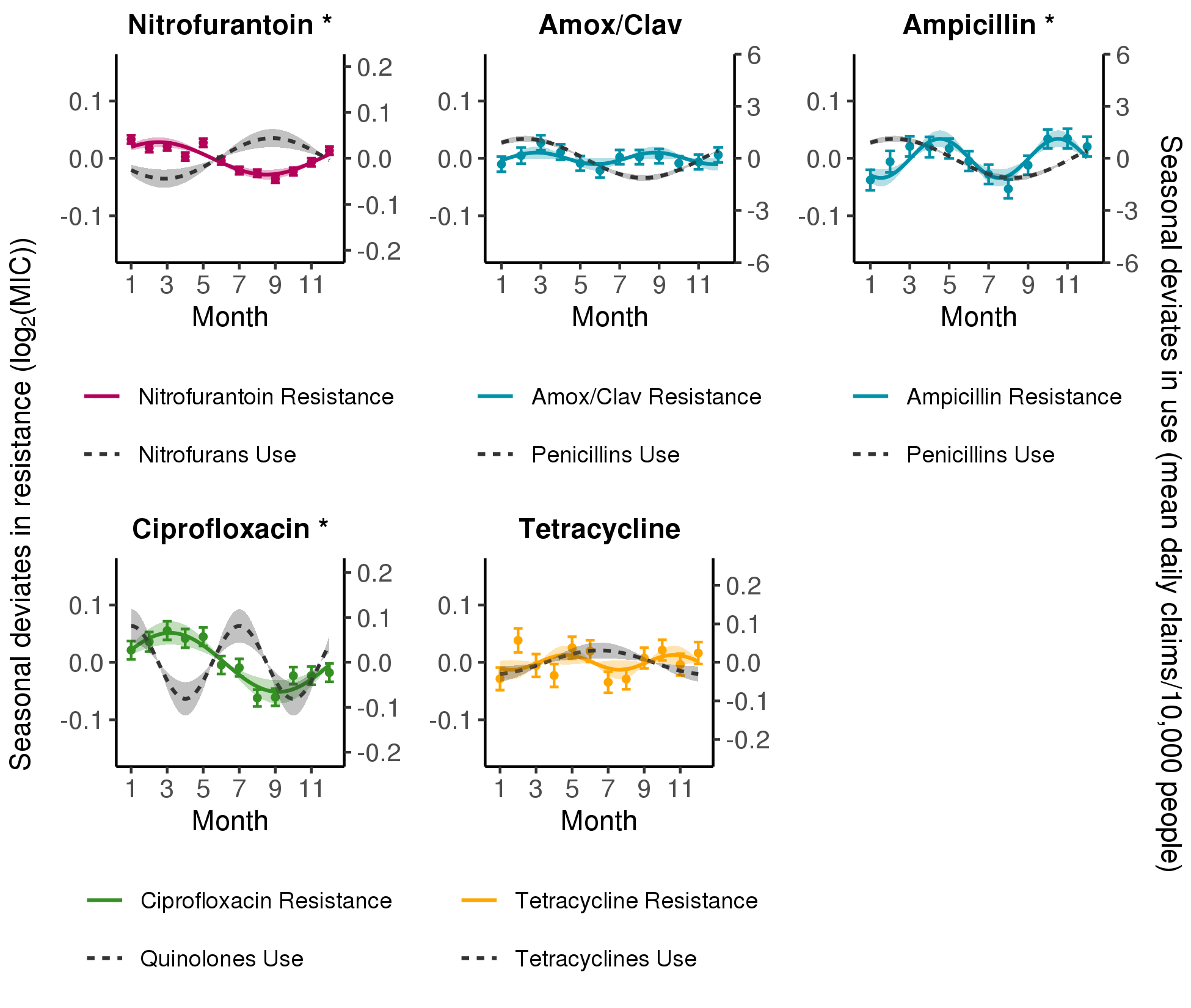

Supplement: S1 Fig — Solid lines indicate point estimates of the amplitude and phase from the best-fitting sinusoidal model of resistance (comparing 6- and 12-month periods) to each antibiotic, colored by class. Dashed gray lines indicate point estimates of the amplitude and phase from sinusoidal models of use of the corresponding antibiotic class. Shaded regions indicate the 95% CIs for the amplitude. Points indicate the monthly mean seasonal deviates in resistance, and error bars indicate the standard error of the mean. Asterisks indicate the amplitude of seasonality in resistance is statistically significant (FDR < 0.05). Amox/Clav, amoxicillin-clavulanate; FDR, false discovery rateS. Underlying data are available at https://github.com/gradlab/use-resistance-seasonality/tree/master/figure_data/S1_Fig [16]. (TIFF) [file pbio.3001579.s001.tiff]

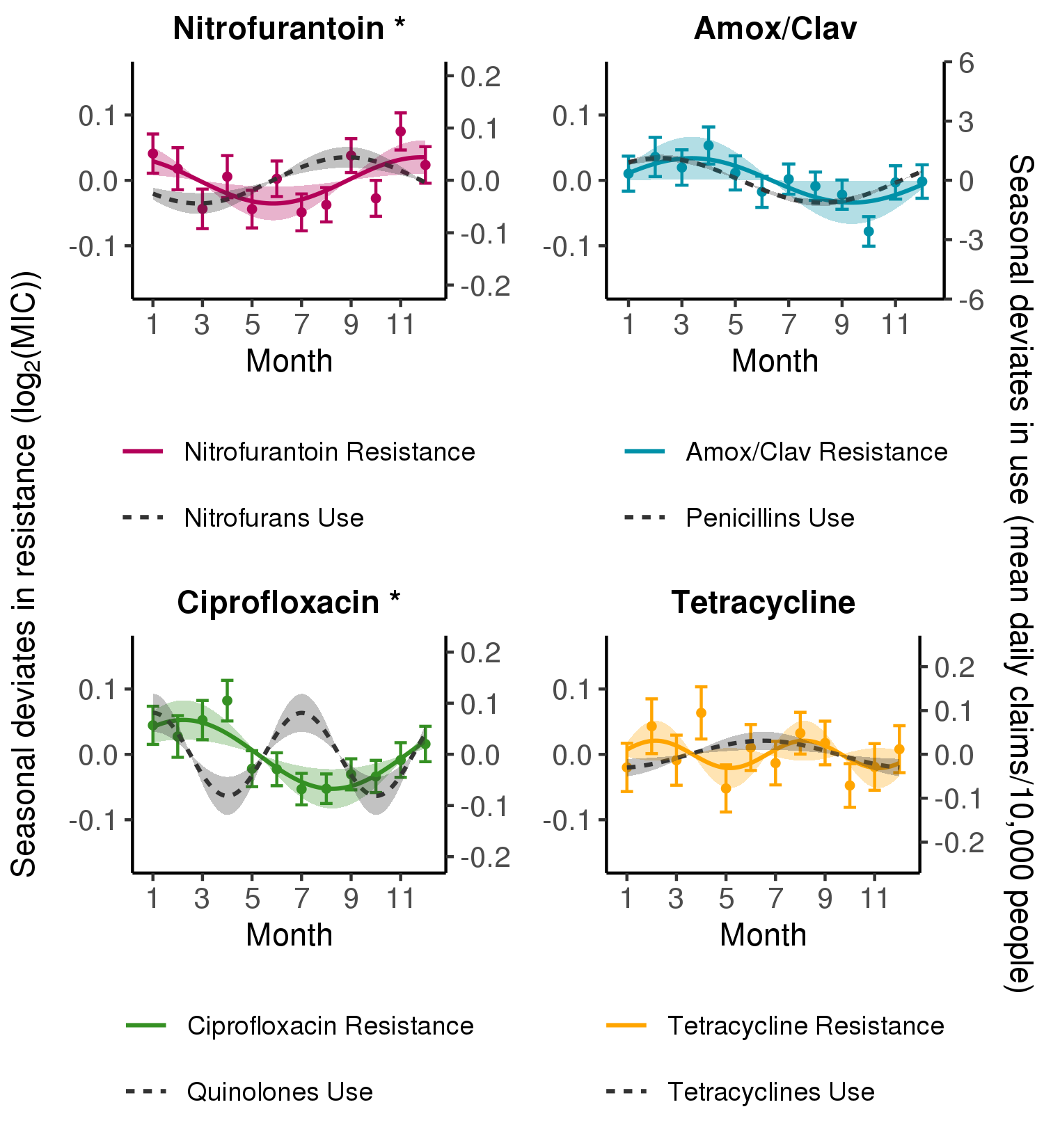

Supplement: S2 Fig — Solid lines indicate point estimates of the amplitude and phase from the best-fitting sinusoidal model of resistance (comparing 6- and 12-month periods) to each antibiotic, colored by class. Dashed gray lines indicate point estimates of the amplitude and phase from sinusoidal models of use of the corresponding antibiotic class. Shaded regions indicate the 95% CIs for the amplitude. Points indicate the monthly mean seasonal deviates in resistance, and error bars indicate the standard error of the mean. Asterisks indicate the amplitude of seasonality in resistance is statistically significant (FDR < 0.05). Amox/Clav, amoxicillin-clavulanate; FDR, false discovery rate. Underlying data are available at https://github.com/gradlab/use-resistance-seasonality/tree/master/figure_data/S2_Fig [16]. (TIFF) [file pbio.3001579.s002.tiff]

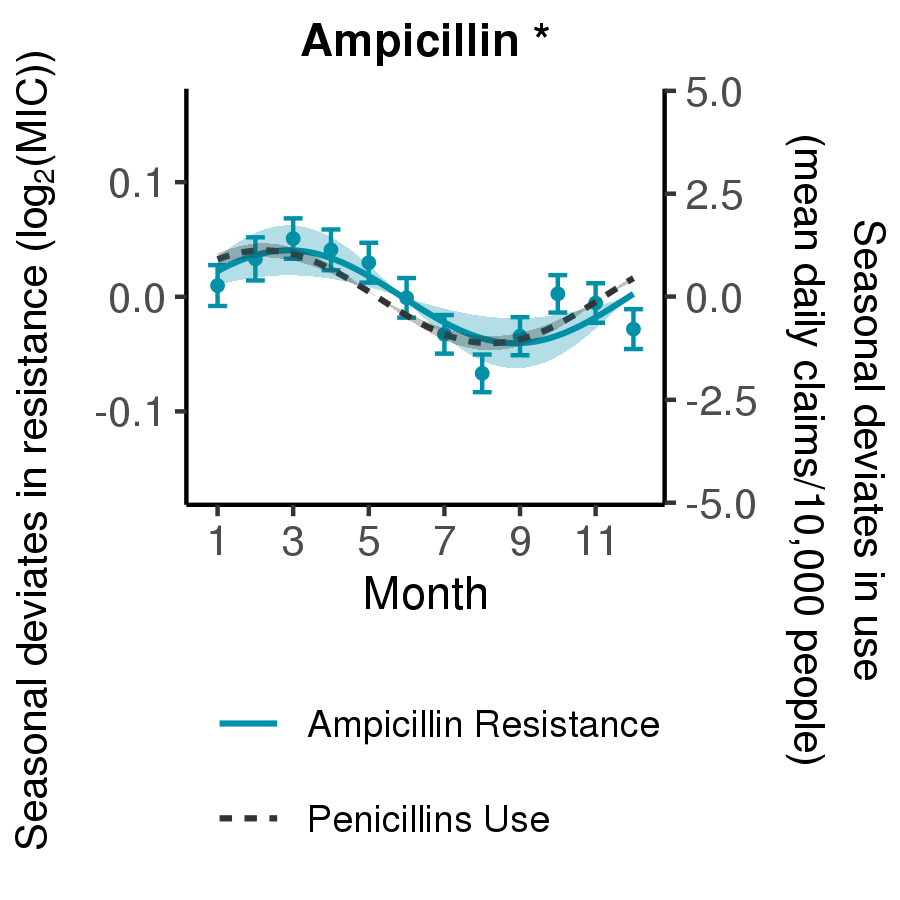

Supplement: S3 Fig — Solid line indicates point estimates of the amplitude and phase from a 12-month period sinusoidal model of resistance to ampicillin in E. coli. Dashed gray line indicates point estimates of the amplitude and phase from a 12-month period sinusoidal model of use of penicillin class antibiotics. Shaded regions indicate the 95% CIs for the amplitude. Points indicate the monthly mean seasonal deviates in resistance, and error bars indicate the standard error of the mean. Asterisk indicates the amplitude of seasonality in resistance is statistically significant (FDR < 0.05). FDR, false discovery rate. Underlying data are available at https://github.com/gradlab/use-resistance-seasonality/tree/master/figure_data/S3_Fig [16]. (TIFF) [file pbio.3001579.s003.tiff]

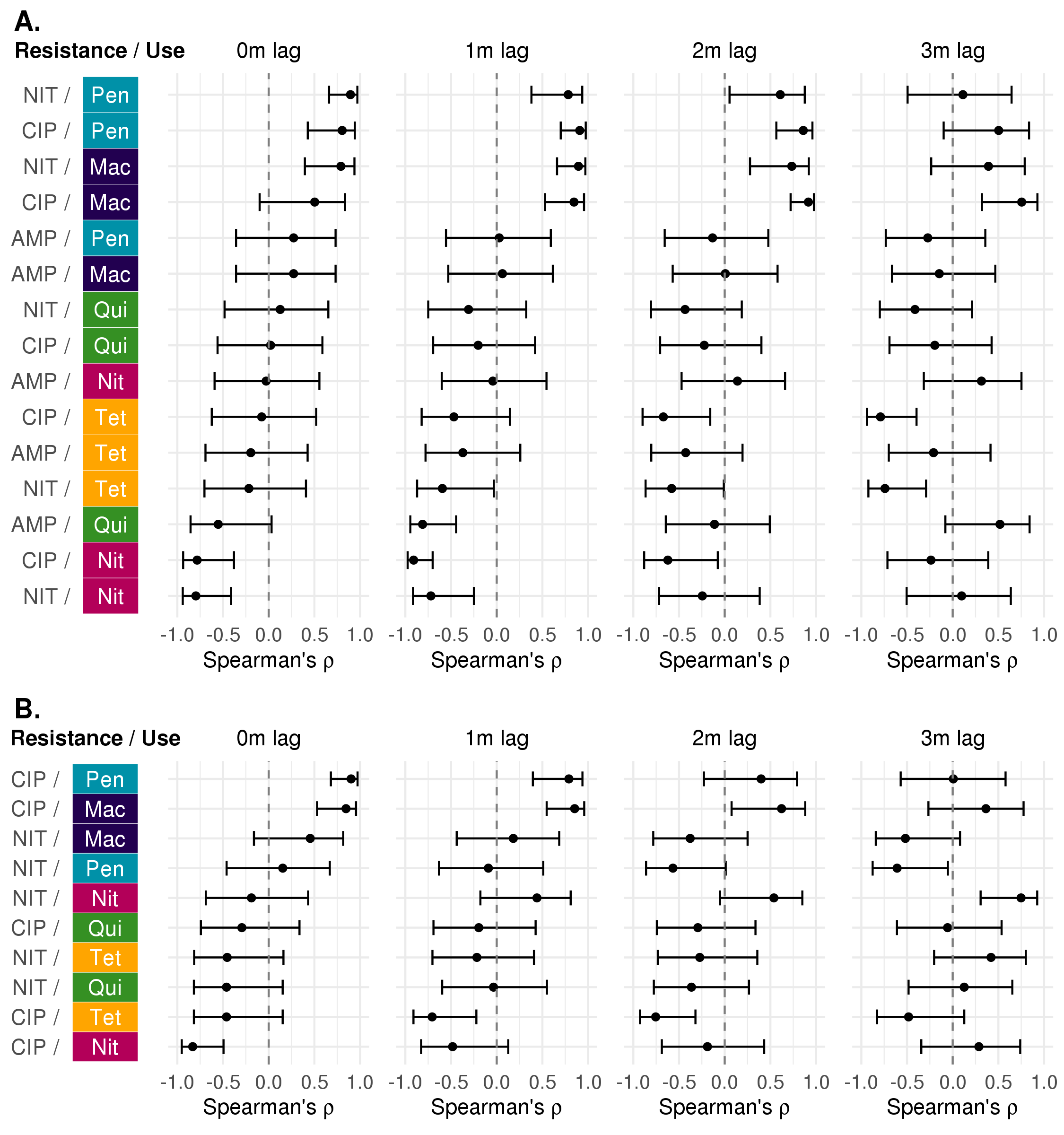

Supplement: S4 Fig — Spearman correlations between seasonal use and resistance with 0 to 3 months lag in (A) E. coli and (B) K. pneumoniae. Spearman rank correlation coefficients were calculated between the monthly mean seasonal deviate in resistance (in log2 (MIC)) and the monthly mean seasonal deviate in use (in average daily claims per 10,000 people) with 0, 1, 2, or 3 months lag between use and resistance, for each pairwise combination of antibiotics and classes. Error bars indicate the 95% CIs. Colors indicate the use antibiotic class. AMP, ampicillin; CIP, ciprofloxacin; Mac, macrolide; MIC, minimum inhibitory concentration; Nit, nitrofuran; NIT, nitrofurantoin; Pen, penicillin; Qui, quinolone; Tet, tetracycline. Underlying data are available at https://github.com/gradlab/use-resistance-seasonality/tree/master/tables/correlations.csv [16]. (TIF) [file pbio.3001579.s004.tif]

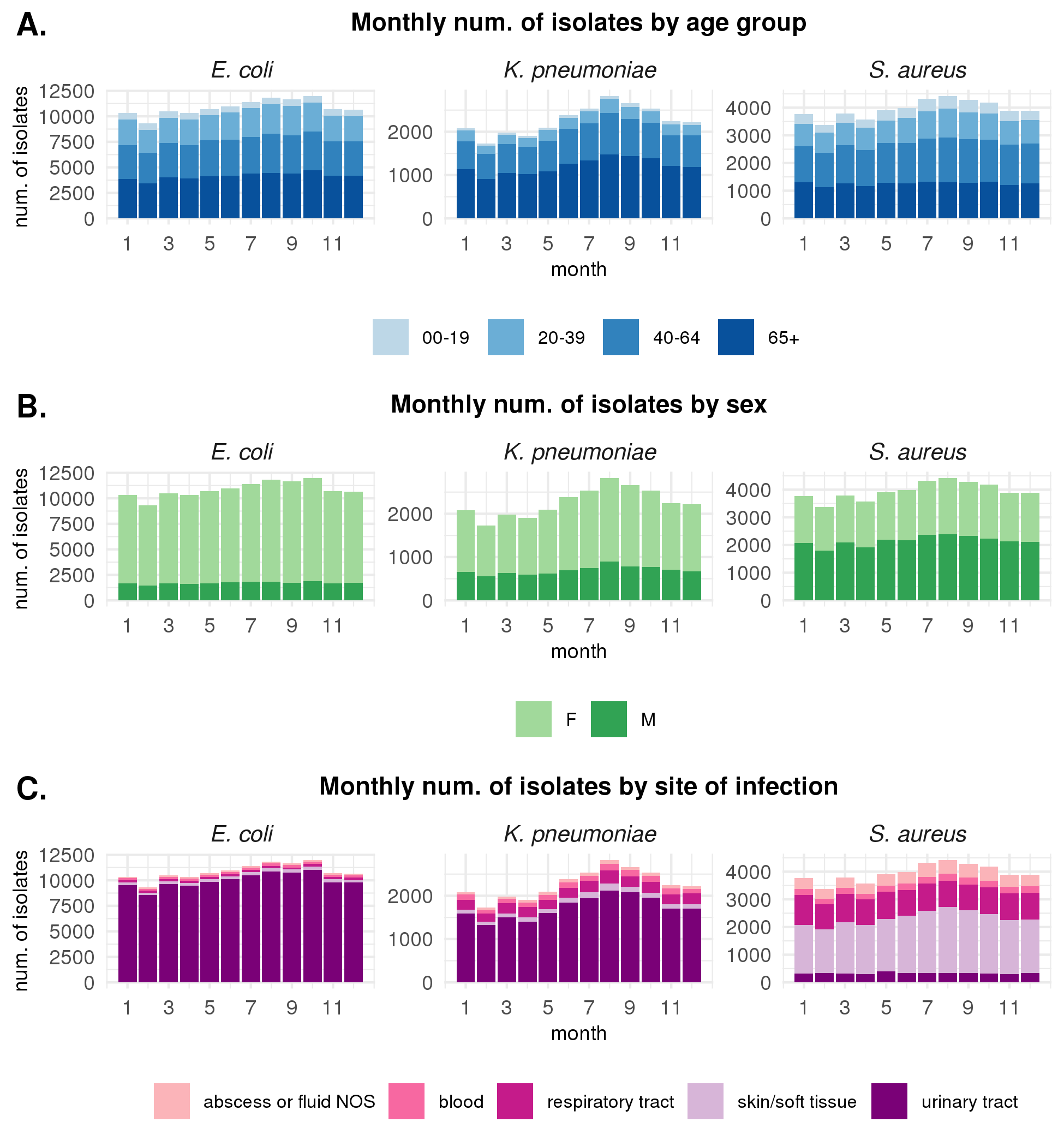

Supplement: S5 Fig — Bars show the total number of isolates by month included in the resistance dataset for each species, colored by (A) age group, (B) sex, and (C) site of infection. NOS, not otherwise specified. Underlying data are available at https://github.com/gradlab/use-resistance-seasonality/tree/master/figure_data/S5_Fig [16]. (TIF) [file pbio.3001579.s005.tif]

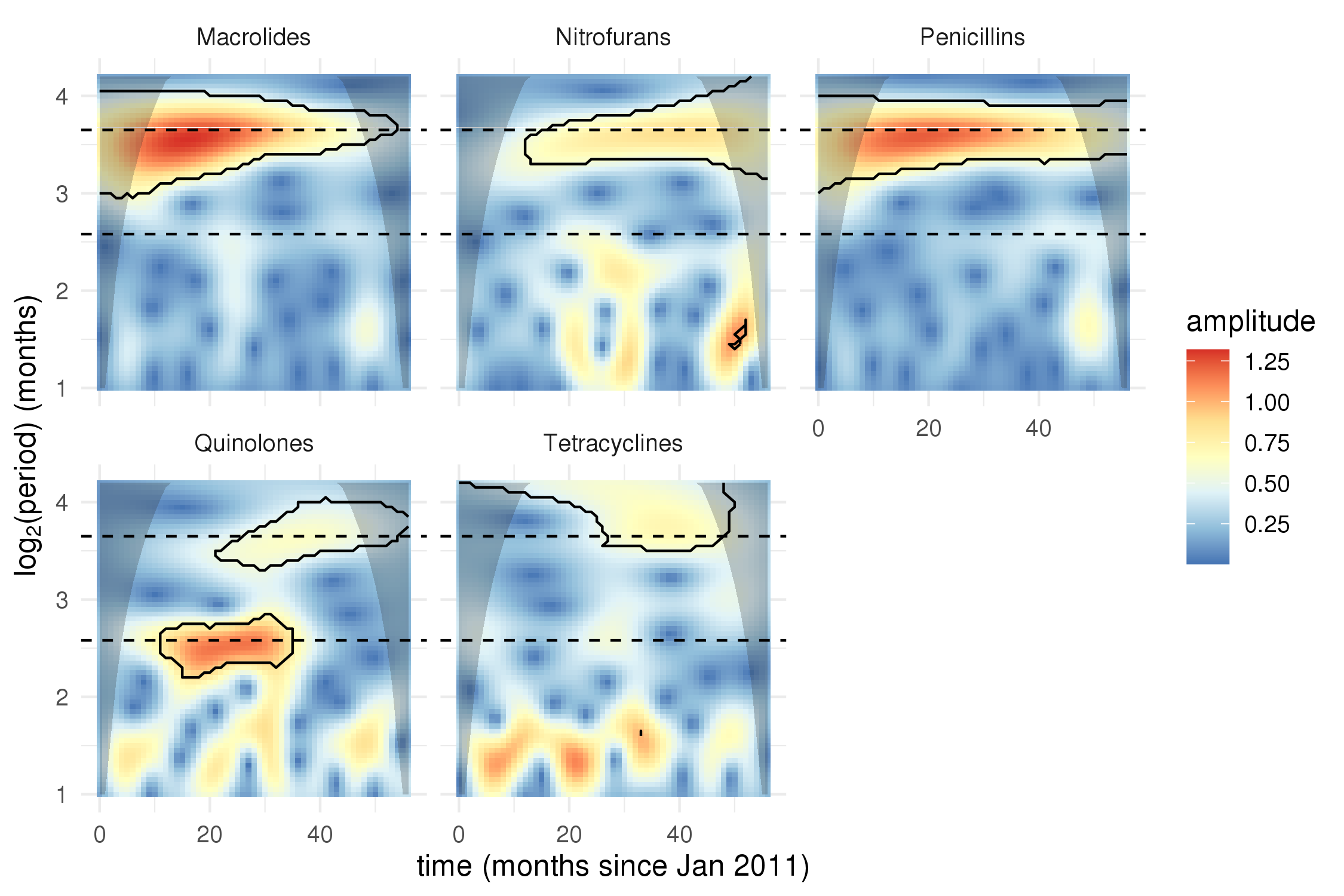

Supplement: S6 Fig — Dotted lines show 12-month (upper line) and 6-month (lower line) periods. Solid lines indicate regions where the amplitude p-value is less than 0.05. Shaded areas indicate the “cone of influence” where edge effects are important. Underlying data are available at https://github.com/gradlab/use-resistance-seasonality/tree/master/figure_data/S6_Fig [16]. (TIFF) [file pbio.3001579.s006.tiff]
