# Supplementary material for: Analysis of multiple bacterial species and antibiotic classes reveals large variation in the association between seasonal antibiotic use and resistance
Source: PLoS Biol. 2022 Mar 9;20(3):e3001579. doi: 10.1371/journal.pbio.3001579 (PMC8936496; doi:10.1371/journal.pbio.3001579)
Supplement: S1 Table — Amplitudes and phases were estimated from the best-fitting sinusoidal model of resistance (comparing 6- and 12-month periods) for each species–antibiotic combination, using data that was subset to include only outpatients under age 65. Asterisks indicate that the amplitude is significant after Benjamini–Hochberg multiple testing correction (FDR < 0.05). AMC, amoxicillin-clavulanate; AMP, ampicillin; CIP, ciprofloxacin; ERY, erythromycin; FDR, false discovery rate; NIT, nitrofurantoin; OXA, oxacillin; PEN, penicillin; TET, tetracycline. (DOCX) [file pbio.3001579.s007.docx]

| **Species** | **Antibiotic** | **Period** | **Amplitude (95% CI)** | **Phase (95% CI)** |
| --- | --- | --- | --- | --- |
| *E. coli* | AMC | 6 months | 0.013 (-1.7e-03, 0.027) | 2.7 (1.6, 3.7) |
| *E. coli* | AMP | 6 months | 0.039 (0.019, 0.06) * | 4.3 (3.8, 4.8) |
| *E. coli* | CIP | 6 months | 0.022 (5.9e-03, 0.037) * | 4.6 (3.9, 5.2) |
| *E. coli* | NIT | 12 months | 0.033 (0.022, 0.044) * | 2.6 (2.1, 3.1) |
| *E. coli* | TET | 6 months | 0.013 (-7.9e-03, 0.034) | 3.6 (2, 5.3) |
| *K. pneumoniae* | AMC | 12 months | 0.028 (-0.027, 0.082) | 4 (1.6, 6.4) |
| *K. pneumoniae* | CIP | 6 months | 0.023 (-0.014, 0.059) | 4.8 (3.4, 6.2) |
| *K. pneumoniae* | NIT | 6 months | 0.027 (-0.013, 0.067) | 5.5 (4.1, 7) |
| *K. pneumoniae* | TET | 12 months | 0.062 (-0.012, 0.14) | 8.3 (6.2, 10) |
| *S. aureus* | CIP | 12 months | 0.043 (2.2e-03, 0.085) | 2.2 (0.46, 3.9) |
| *S. aureus* | ERY | 6 months | 0.056 (8.9e-03, 0.1) | 2.6 (1.8, 3.4) |
| *S. aureus* | NIT | 12 months | 0.041 (0.025, 0.058) * | 2.4 (1.7, 3.1) |
| *S. aureus* | OXA | 12 months | 0.03 (-0.017, 0.078) | 11 (8.2, 13) |
| *S. aureus* | PEN | 12 months | 0.031 (-5.7e-03, 0.069) | 9.1 (7.5, 11) |
| *S. aureus* | TET | 12 months | 0.019 (-8.6e-03, 0.047) | 11 (8.4, 13) |
