# Supplementary material for: Analysis of multiple bacterial species and antibiotic classes reveals large variation in the association between seasonal antibiotic use and resistance
Source: PLoS Biol. 2022 Mar 9;20(3):e3001579. doi: 10.1371/journal.pbio.3001579 (PMC8936496; doi:10.1371/journal.pbio.3001579)
Supplement: S2 Table — Demographic and site of infection coefficients were estimated from the sinusoidal model of resistance that was adjusted for patient age, sex, and site of infection (Eq 4 in Materials and methods). In parentheses are the 95% CIs on the coefficient estimates. Asterisks indicate that the coefficient is significant after Benjamini–Hochberg multiple testing correction (FDR < 0.05). βa, coefficient for patient age; βs, coefficient for patient sex; βbl, coefficient for if the isolate was a blood isolate; βrt, coefficient for if the isolate was a respiratory tract isolate; βsst, coefficient for if the isolate was a skin/soft tissue isolate; βab, coefficient for if the isolate was an abscess/fluid isolate; AMC, amoxicillin-clavulanate; AMP, ampicillin; CIP, ciprofloxacin; ERY, erythromycin; FDR, false discovery rate; NIT, nitrofurantoin; OXA, oxacillin; PEN, penicillin; TET, tetracycline. (DOCX) [file pbio.3001579.s008.docx]

| **Species** | **Abx** | **Period** | $\boldsymbol{\beta}_{\boldsymbol{a}}$ | $\boldsymbol{\beta}_{\boldsymbol{s}}$ | $\boldsymbol{\beta}_{\boldsymbol{bl}}$ | $\boldsymbol{\beta}_{\boldsymbol{rt}}$ | $\boldsymbol{\beta}_{\boldsymbol{sst}}$ | $\boldsymbol{\beta}_{\boldsymbol{ab}}$ |
| --- | --- | --- | --- | --- | --- | --- | --- | --- |
| *E. coli* | AMC | 6 months | 2.0e-03 (1.7e-03, 2.3e-03) * | 0.25 (0.23, 0.27) * | 0.23 (0.17, 0.28) * | 0.35 (0.3, 0.41) * | 0.045  (-2.6e-03, 0.093) | 0.12 (0.059, 0.18) * |
| *E. coli* | AMP | 6 months | 1.9e-03 (1.5e-03, 2.3e-03) * | 0.34 (0.32, 0.37) * | 0.22 (0.15,  0.3) * | 0.43 (0.36, 0.51) * | -4.3e-04  (-0.063, 0.063) | 0.062  (-0.019, 0.14) |
| *E. coli* | CIP | 12 months | 0.013 (0.013, 0.014) * | 0.54 (0.51, 0.56) * | 0.4 (0.34, 0.47) * | 0.64 (0.58, 0.7) * | 0.11 (0.056, 0.17) * | 0.099 (0.027, 0.17) * |
| *E. coli* | NIT | 12 months | 7.6e-04 (5.8e-04, 9.3e-04) * | 0.053 (0.042, 0.065) * | -0.05  (-0.08,  -0.02) * | -0.091  (-0.12,  -0.063) * | -0.05  (-0.077,  -0.023) * | -0.077  (-0.11,  -0.043) * |
| *E. coli* | TET | 6 months | 1.1e-03 (6.0e-04, 1.5e-03) * | 0.3 (0.27, 0.33) * | 0.34 (0.26, 0.42) * | 0.28 (0.2, 0.36) * | 0.04  (-0.031, 0.11) | -0.056  (-0.15, 0.035) |
| *K. pneumoniae* | AMC | 12 months | -8.2e-04  (-1.5e-03,  -1.1e-04) * | 0.24 (0.21, 0.27) * | 0.13 (0.065, 0.2) * | 0.29 (0.24, 0.34) * | 0.14 (0.078, 0.21) * | 0.16 (0.077, 0.25) * |
| *K. pneumoniae* | CIP | 12 months | 8.0e-04 (3.4e-05, 1.6e-03) * | 0.4 (0.37, 0.44) * | 0.079 (9.0e-03, 0.15) * | 0.27 (0.22, 0.32) * | -0.041  (-0.11, 0.03) | 0.044  (-0.044, 0.13) |
| *K. pneumoniae* | NIT | 12 months | -1.2e-03  (-2.0e-03,  -3.9e-04) * | 0.18 (0.15, 0.22) * | 0.08 (3.7e-03, 0.16) * | 0.11 (0.056, 0.17) * | -3.7e-03  (-0.085, 0.077) | 0.063  (-0.038, 0.16) |
| *K. pneumoniae* | TET | 6 months | -2.7e-03  (-3.7e-03,  -1.6e-03) * | 0.25 (0.21, 0.3) * | 5.0e-03  (-0.091, 0.1) | 0.18 (0.11, 0.25) * | 0.064  (-0.032, 0.16) | 0.023  (-0.099, 0.14) |
| *S. aureus* | CIP | 12 months | 0.016 (0.015, 0.017) * | 0.02  (-0.011, 0.051) | -0.79  (-0.87,  -0.71) * | -0.34  (-0.4,  -0.28) * | -1.1  (-1.1, -1) * | -0.9  (-0.97,  -0.83) * |
| *S. aureus* | ERY | 12 months | 4.9e-03 (3.9e-03, 5.9e-03) * | -0.079  (-0.12,  -0.035) * | -0.41  (-0.53,  -0.29) * | -0.031  (-0.12, 0.056) | -0.57  (-0.66,  -0.49) * | -0.049  (-0.15, 0.053) |
| *S. aureus* | NIT | 12 months | -7.5e-06  (-2.4e-04, 2.2e-04) | -8.1e-04 (-0.011, 9.9e-03) | 0.21 (0.18, 0.24) * | 0.076 (0.056, 0.097) * | 0.22 (0.2, 0.24) * | 0.25 (0.22, 0.27) * |
| *S. aureus* | OXA | 12 months | 4.6e-03 (3.9e-03, 5.3e-03) * | -3.9e-03 (-0.034, 0.027) | -0.39  (-0.47,  -0.3) * | -0.19  (-0.25,  -0.13) * | -0.54  (-0.6, -0.48) * | 0.026  (-0.045, 0.096) |
| *S. aureus* | PEN | 6 months | -9.4e-04  (-1.4e-03,  -4.5e-04) * | -2.9e-04 (-0.022, 0.022) | -0.034  (-0.093, 0.026) | 0.036  (-7.9e-03, 0.08) | -0.025  (-0.066, 0.017) | 0.093 (0.042, 0.14) * |
| *S. aureus* | TET | 12 months | -3.1e-04  (-7.1e-04, 9.2e-05) | -0.013 (-0.03, 5.3e-03) | -0.058  (-0.11, -9.5e-03) * | -0.055  (-0.09, -0.02) * | -0.025  (-0.059, 8.0e-03) | -0.09  (-0.13,  -0.048) * |
