# Supplementary material for: Analysis of multiple bacterial species and antibiotic classes reveals large variation in the association between seasonal antibiotic use and resistance
Source: PLoS Biol. 2022 Mar 9;20(3):e3001579. doi: 10.1371/journal.pbio.3001579 (PMC8936496; doi:10.1371/journal.pbio.3001579)
Supplement: S3 Table — Antibiotic claims data for Boston, Massachusetts residents was obtained from the Massachusetts All Payers Claims Database and subset by antibiotic class according to the following WHO ATC codes [42]: penicillins, J01C; macrolides, J01F; quinolones, J01M; tetracyclines, J01A; and nitrofurans, J01XE. All antibiotics under each ATC header that were present in this dataset were included in the analysis. The percentage of the total claims within each antibiotic class made up by each individual antibiotic is listed in the last column of the table. (DOCX) [file pbio.3001579.s009.docx]

| **Antibiotic class** | **Antibiotic name** | **ATC code** | **% of total use within antibiotic class** |
| --- | --- | --- | --- |
| Penicillins | Amoxicillin | J01CA04 | 83 |
|  | Phenoxymethylpenicillin | J01CE02 | 16 |
|  | Dicloxacillin | J01CF01 | 1.1 |
|  | Ampicillin | J01CA01 | 0.35 |
|  | Benzylpenicillin | J01CE01 | 0.091 |
|  | Piperacillin | J01CA12 | 0.036 |
|  | Nafcillin | J01CF06 | 0.024 |
|  | Oxacillin | J01CF04 | 3.0e-03 |
| Macrolides | Azithromycin | J01FA10 | 51 |
|  | Clindamycin | J01FF01 | 28 |
|  | Erythromycin | J01FA01 | 17 |
|  | Clarithromycin | J01FA09 | 3.8 |
|  | Lincomycin | J01FF02 | 1.1e-03 |
|  | Telithromycin | J01FA15 | 7.0e-04 |
| Quinolones | Ciprofloxacin | J01MA02 | 67 |
|  | Levofloxacin | J01MA12 | 14 |
|  | Ofloxacin | J01MA01 | 13 |
|  | Moxifloxacin | J01MA14 | 5.7 |
|  | Gatifloxacin | J01MA16 | 0.85 |
|  | Norfloxacin | J01MA06 | 0.071 |
|  | Gemifloxacin | J01MA15 | 9.8e-03 |
| Tetracyclines | Doxycycline | J01AA02 | 79 |
|  | Minocycline | J01AA08 | 19 |
|  | Tetracycline | J01AA07 | 2 |
|  | Tigecycline | J01AA12 | 0.054 |
|  | Demeclocycline | J01AA01 | 0.053 |
| Nitrofurans | Nitrofurantoin | J01XE01 | 100 |
