# Supplementary material for: Analysis of multiple bacterial species and antibiotic classes reveals large variation in the association between seasonal antibiotic use and resistance
Source: PLoS Biol. 2022 Mar 9;20(3):e3001579. doi: 10.1371/journal.pbio.3001579 (PMC8936496; doi:10.1371/journal.pbio.3001579)
Supplement: S4 Table — Table of the total number of isolates of each species within each clinical or demographic category that was used in this analysis. In parentheses is the percent of the total number of isolates for that species. BWH, Brigham and Women’s Hospital; MGH, Massachusetts General Hospital; NOS, not otherwise specified. (DOCX) [file pbio.3001579.s010.docx]

|  |  | ***E. coli*** | ***K. pneumoniae*** | ***S. aureus*** |
| --- | --- | --- | --- | --- |
| Total | Total | 130407 | 27178 | 47374 |
| Hospitals | BWH | 53428 (41%) | 11716 (43.1%) | 17741 (37.4%) |
|  | MGH | 76979 (59%) | 15462 (56.9%) | 29633 (62.6%) |
| Patient type | Inpatient | 23146 (17.7%) | 9498 (34.9%) | 16897 (35.7%) |
|  | Outpatient | 107261 (82.3%) | 17680 (65.1%) | 30477 (64.3%) |
| Site of infection | Abscess or fluid NOS | 1971 (1.5%) | 806 (3%) | 5152 (10.9%) |
|  | Blood | 2617 (2%) | 1419 (5.2%) | 2802 (5.9%) |
|  | Respiratory tract | 2643 (2%) | 2859 (10.5%) | 11598 (24.5%) |
|  | Skin and soft tissue | 3314 (2.5%) | 1324 (4.9%) | 23841 (50.3%) |
|  | Urinary tract | 119862 (91.9%) | 20770 (76.4%) | 3981 (8.4%) |
| Age group | 00-19 | 7629 (5.9%) | 708 (2.6%) | 4455 (9.4%) |
|  | 20-39 | 31247 (24%) | 3003 (11%) | 10496 (22.2%) |
|  | 40-64 | 41591 (31.9%) | 8977 (33%) | 17312 (36.5%) |
|  | 65+ | 49940 (38.3%) | 14490 (53.3%) | 15111 (31.9%) |
| Sex | Female | 109948 (84.3%) | 18827 (69.3%) | 21565 (45.5%) |
|  | Male | 20459 (15.7%) | 8351 (30.7%) | 25809 (54.5%) |
