# Supplementary material for: Analysis of multiple bacterial species and antibiotic classes reveals large variation in the association between seasonal antibiotic use and resistance
Source: PLoS Biol. 2022 Mar 9;20(3):e3001579. doi: 10.1371/journal.pbio.3001579 (PMC8936496; doi:10.1371/journal.pbio.3001579)
Supplement: S5 Table — Percent resistance was calculated as the percentage of nonsusceptible isolates out of the total number of isolates from each hospital with a reported MIC for that antibiotic. BWH, Brigham and Women’s Hospital; MGH, Massachusetts General Hospital; MIC, minimum inhibitory concentration. (DOCX) [file pbio.3001579.s011.docx]

| **Species** | **Antibiotic (Antibiotic class)** | **Dates included at BWH** | **Dates included at MGH** | **Percent resistance at BWH** | **Percent resistance at MGH** |
| --- | --- | --- | --- | --- | --- |
| *E. coli* | Amoxicillin/  Clavulanate (Penicillins) | May 2013 – Dec 2019 | Jan 2007 – Dec 2016 | 17.8% | 17.2% |
|  | Ampicillin (Penicillins) | Jan 2007 – Dec 2019 | Jan 2007 – Dec 2016 | 47.8% | 47.0% |
|  | Ciprofloxacin (Quinolones) | Jan 2007 – Dec 2019 | Jan 2007 – Dec 2016 | 22.6% | 21.1% |
|  | Nitrofurantoin (Nitrofurans) | Jan 2007 – Jun 2013 | Jan 2007 – Dec 2016 | 6.0% | 5.1% |
|  | Tetracycline (Tetracyclines) | May 2013 – Dec 2019 | Jan 2007 – Dec 2016 | 29.1% | 29.3% |
| *K. pneumoniae* | Amoxicillin/  Clavulanate (Penicillins) | May 2013 – Dec 2019 | Jan 2007 – Dec 2016 | 10.2% | 8.2% |
|  | Ciprofloxacin (Quinolones) | Jan 2007 – Dec 2019 | Jan 2007 – Dec 2016 | 11.8% | 11.1% |
|  | Nitrofurantoin (Nitrofurans) | Jan 2007 – Jun 2013 | Jan 2007 – Dec 2016 | 69.4% | 68.0% |
|  | Tetracycline (Tetracyclines) | May 2013 – Dec 2019 | Jan 2007 – Dec 2016 | 21.3% | 18.3% |
| *S. aureus* | Ciprofloxacin (Quinolones) | May 2010 – Dec 2019 | Jan 2009 – Dec 2016 | 29.6% | 30.7% |
|  | Erythromycin (Macrolides) | May 2010 – Dec 2019 | Jan 2009 – Dec 2016 | 51.2% | 54.9% |
|  | Nitrofurantoin (Nitrofurans) | May 2010 – Jun 2013 | Jan 2009 – Dec 2016 | 1.1% | 1.0% |
|  | Oxacillin (Penicillins) | May 2010 – Dec 2019 | Jan 2009 – Dec 2016 | 33.4% | 35.9% |
|  | Penicillin (Penicillins) | May 2010 – Dec 2019 | Jan 2009 – Apr 2015 | 82.4% | 84.3% |
|  | Tetracycline (Tetracyclines) | May 2010 – Dec 2019 | Jan 2009 – Dec 2016 | 6.6% | 6.0% |
