# Supplementary material for: Analysis of multiple bacterial species and antibiotic classes reveals large variation in the association between seasonal antibiotic use and resistance
Source: PLoS Biol. 2022 Mar 9;20(3):e3001579. doi: 10.1371/journal.pbio.3001579 (PMC8936496; doi:10.1371/journal.pbio.3001579)
Supplement: S6 Table — In parentheses is the difference in AIC from the model with the lower AIC. AIC, Akaike information criterion. (DOCX) [file pbio.3001579.s012.docx]

| **Antibiotic class** | **Antibiotic Use Regression Model** | |
| --- | --- | --- |
|  | **6-month period** | **12-month period** |
| Macrolides | 104.4 (+67.5) | 36.9 (+0) |
| Nitrofurans | -201.0 (+12.1) | -213.1 (+0) |
| Penicillins | 128.7 (+86.6) | 42.0 (+0) |
| Quinolones | -89.5 (+0) | -79.6 (+9.9) |
| Tetracyclines | -155.1 (+6.1) | -161.2 (+0) |
