# Supplementary material for: Analysis of multiple bacterial species and antibiotic classes reveals large variation in the association between seasonal antibiotic use and resistance
Source: PLoS Biol. 2022 Mar 9;20(3):e3001579. doi: 10.1371/journal.pbio.3001579 (PMC8936496; doi:10.1371/journal.pbio.3001579)
Supplement: S7 Table — In parentheses is the difference in AIC from the model with the lower AIC. AIC, Akaike information criterion. (DOCX) [file pbio.3001579.s013.docx]

| **Species** | **Antibiotic** | **Antibiotic Resistance Regression Model** | |
| --- | --- | --- | --- |
|  |  | **6-month period** | **12-month period** |
| *E. coli* | Amoxicillin/Clavulanate | 339247.4 (+0) | 339249.8 (+2.4) |
|  | Ampicillin | 522468.9 (+0) | 522474.6 (+5.8) |
|  | Ciprofloxacin | 498206.9 (+7.9) | 498199 (+0) |
|  | Nitrofurantoin | 199978.2 (+21.3) | 199956.9 (+0) |
|  | Tetracycline | 422473.8 (+0) | 422474.0 (+0.2) |
| *K. pneumoniae* | Amoxicillin/Clavulanate | 64714.5 (+3.8) | 64710.6 (+0) |
|  | Ciprofloxacin | 89697.3 (+11.1) | 89686.2 (+0) |
|  | Nitrofurantoin | 66945.1 (+7.5) | 66937.6 (+0) |
|  | Tetracycline | 80426.2 (+0) | 80427.8 (+1.6) |
| *S. aureus* | Ciprofloxacin | 189787.4 (+21.6) | 189765.8 (+0) |
|  | Erythromycin | 216426.9 (+5.1) | 216421.7 (+0) |
|  | Nitrofurantoin | 52484.0 (+15.6) | 52468.4 (+0) |
|  | Oxacillin | 183142.1 (+7.5) | 183134.6 (+0) |
|  | Penicillin | 125212.3 (+0) | 125212.5 (+0.2) |
|  | Tetracycline | 131731.2 (+1.5) | 131729.7 (+0) |
